# 17.1% Efficient Single‐Junction Organic Solar Cells Enabled by n‐Type Doping of the Bulk‐Heterojunction

**DOI:** 10.1002/advs.201903419

**Published:** 2020-02-13

**Authors:** Yuanbao Lin, Yuliar Firdaus, Mohamad Insan Nugraha, Feng Liu, Safakath Karuthedath, Abdul‐Hamid Emwas, Weimin Zhang, Akmaral Seitkhan, Marios Neophytou, Hendrik Faber, Emre Yengel, Iain McCulloch, Leonidas Tsetseris, Frédéric Laquai, Thomas D. Anthopoulos

**Affiliations:** ^1^ King Abdullah University of Science and Technology (KAUST) KAUST Solar Center (KSC) Thuwal 23955 Saudi Arabia; ^2^ Department of Polymer Science and Engineering School of Chemistry and Chemical Engineering Shanghai Jiao Tong University Shanghai 200240 P. R. China; ^3^ King Abdullah University of Science and Technology (KAUST) Core Labs Thuwal 23955 Saudi Arabia; ^4^ Department of Physics National Technical University of Athens Athens GR‐15780 Greece

**Keywords:** additives, molecular doping, nonfullerene acceptors, organic photovoltaics

## Abstract

Molecular doping is often used in organic semiconductors to tune their (opto)electronic properties. Despite its versatility, however, its application in organic photovoltaics (OPVs) remains limited and restricted to p‐type dopants. In an effort to control the charge transport within the bulk‐heterojunction (BHJ) of OPVs, the n‐type dopant benzyl viologen (BV) is incorporated in a BHJ composed of the donor polymer PM6 and the small‐molecule acceptor IT‐4F. The power conversion efficiency (PCE) of the cells is found to increase from 13.2% to 14.4% upon addition of 0.004 wt% BV. Analysis of the photoactive materials and devices reveals that BV acts simultaneously as n‐type dopant and microstructure modifier for the BHJ. Under optimal BV concentrations, these synergistic effects result in balanced hole and electron mobilities, higher absorption coefficients and increased charge‐carrier density within the BHJ, while significantly extending the cells' shelf‐lifetime. The n‐type doping strategy is applied to five additional BHJ systems, for which similarly remarkable performance improvements are obtained. OPVs of particular interest are based on the ternary PM6:Y6:PC_71_BM:BV(0.004 wt%) blend for which a maximum PCE of 17.1%, is obtained. The effectiveness of the n‐doping strategy highlights electron transport in NFA‐based OPVs as being a key issue.

## Graphical abstract

Addition of the n‐type dopant benzyl viologen (BV) into several best‐in‐class organic bulk‐heterojunctions (BHJ) is shown to consistently improve the power conversion efficiency (PCE) of the resulting solar cells. The presence of BV inside the BHJs increases the absorption coefficient, balances charge transport, and enhances the charge‐carrier density. These synergistic effects result in organic photovoltaics with a maximum PCE of 17.1%.

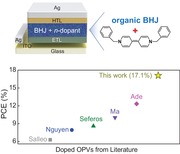

Organic photovoltaics (OPVs) represent a promising solar energy harvesting technology offering numerous attractive attributes that include: light weight and mechanical flexibility with the potential for low manufacturing cost due to their broad compatibility with large‐area processing techniques.^1^ In recent years, most efforts have been dedicated to the design of new materials with improved charge carrier mobility, optimized light absorption characteristics, and carefully tuned bulk heterojunction (BHJ) microstructures. As a result, power conversion efficiency (PCE) values of 17% for single‐junction and well over 17% for tandem OPVs, respectively, have been achieved.^2^, ^3^, ^4^ Despite the impressive progress, however, the aforementioned levels of performance are still lower than the practical PCE limits that are predicted to surpass 20% and 25% for single‐junction and tandem OPV cells, respectively.^5^ Development of new materials with simultaneous optimization of the device engineering represent a few approaches currently being pursued for improving the OPV performance further with ultimate target the aforementioned theoretically predictions.

The intentional introduction of molecular dopants has been used extensively to alter the charge transport properties of organic semiconductors, particularly in the area of organic thin‐film transistors (OTFTs) for which some of the highest carrier mobilities have been achieved via this method.^6^, ^7^, ^8^, ^9^ Molecular doping relies on charge transfer interaction(s) between the dopant and the host semiconductor, which ultimately results in formation of free carriers.^10^, ^11^ Recent studies have shown that molecular doping may, under certain conditions, induce multiple synergistic effects including microstructural changes to the host semiconductor and drastic improvement in charge carrier transport.^6^, ^8^, ^9^ These effects were shown to be the result of the dual functionality of the molecular dopants, acting both as electronic dopants and microstructure modifying agents. Noticeable dopant‐induced effects included enhanced layer crystallinity and reduced trap density due to increased free carrier concentration.^8^, ^9^

In the field of OPVs, intentional p‐type doping of the charge transporting interlayers has been studied and shown to be a viable strategy to enhance the cell's PCE by improving the efficiency of carrier extraction from the photoactive layer.^12^, ^13^, ^14^, ^15^, ^16^ The introduction of molecular dopants directly into the active layer of OPVs, on the other hand, has received significantly less attention, often leading to different conclusions, whilst being limited to p‐type dopants,^17^, ^18^, ^19^, ^20^, ^21^ with only one study reporting the use of n‐type dopants for improving the performance of all‐polymer solar cells.^22^ Irrespective of the exact mechanism and the doping approaches adopted, it is clear that there is plenty of scope to explore more molecular dopants and study their suitability in OPV applications.^23^, ^24^

Motivated by earlier studies on molecular dopants,^8^, ^9^, ^25^, ^26^, ^27^, ^28^ we investigated the possibility of using benzyl viologen (BV) (**Figure** **1**a) as n‐type dopant for the BHJ in OPVs. BV is a known n‐type dopant for a variety of semiconductors, including organic and 2D systems, but it has never been used in OPVs.^29^, ^30^, ^31^, ^32^ Besides, its high solubility in organic solvents enables facile incorporation into the host material(s) from solution phase.^31^ Here, we show that the addition of BV directly into BHJ results in n‐type doping while simultaneously increasing the optical absorption coefficients and device photoresponse. In OPVs based on the binary PM6:IT‐4F^33^ blend, addition of 0.004 wt% of BV leads to a consistent PCE enhancement from 13.2% to 14.4%. We find that the presence of BV within the BHJ hinders aggregation, which in turn results in optimized phase separation, improved molecular packing, and balanced carrier transport/extraction. Increasing the BV concentration to 0.4 wt% leads to a PCE drop (9.1%), an effect ascribed to microstructural changes and increased bimolecular recombination. More importantly, we demonstrate the applicability of BV to five additional BHJ systems, for which equally impressive improvements are achieved. BHJ cells of particular interest are based on the binary PM6:Y6^34^ and ternary PM6:Y6:PC_71_BM^24^ systems (PM6: Poly[(2,6‐(4,8‐bis(5‐(2‐ethylhexyl‐3‐fluoro)thiophen‐2‐yl)‐benzo[1,2‐b:4,5‐b′]dithiophene))‐alt‐(5,5‐(1′,3′‐di‐2‐thienyl‐5′,7′‐bis(2‐ethylhexyl)benzo[1′,2′‐c:4′,5′‐c′]dithiophene‐4,8‐dione)], Y6: 2,2′‐((2Z,2′Z)‐((12,13‐bis(2‐ethylhexyl)‐3,9‐diundecyl‐12,13‐dihydro‐[1,2,5]thiadiazolo[3,4‐e]thieno[2,″30″:4′,5′]thieno[2′,3′:4,5]pyrrolo[3,2‐g]thieno[2′,3′:4,5]thieno[3,2‐b]indole‐2,10‐diyl)bis(methanylylidene))bis(5,6‐difluoro‐3‐oxo‐2,3‐dihydro‐1H‐indene‐2,1‐diylidene))dimalononitrile, and PC_71_BM: [6,6]‐phenyl‐C71‐butyric acid methyl ester), for which maximum PCE values of 16% and 17.1% are obtained, respectively.

**Figure 1:**
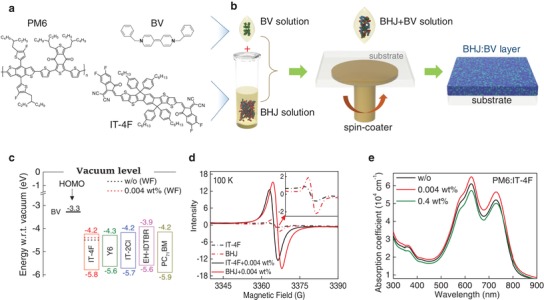
a) The chemical structures of PM6, IT‐4F, and BV. b) Schematic depiction of the process steps used for the preparation of the BV‐doped BHJ layers. c) Energy level diagram of the various materials considered. HOMO energies were obtained directly from the solid layers via PESA. LUMO values are calculated by adding the experimentally measured HOMO and optical bandgap energies of the various materials. The work‐functions (WF) were measured using the Kelvin Probe under inert atmosphere. d) Relative EPR spectra of various layers without (w/o) and with 0.004 wt% BV. e) Absorption coefficient profiles of PM6:IT‐4F blend films doped with different BV concentrations.

Figure 1a shows the chemical structures of the polymer donor PM6, the acceptor IT‐4F, and the BV dopant. Dopant‐containing formulations were prepared by adding the desired amount of BV solution into the neat PM6, IT‐4F, or their PM6:IT‐4F blend, solution, and then left to stir at room temperature for 2 h before spin‐coating (Figure 1b). The BV concentration was calculated as a weight percentage of the solid weight mass of the donor and acceptor materials (see the Experimental Section). To determine whether BV acts as n‐type dopant for the materials considered in this work, the highest occupied molecular orbital (HOMO) energy of each acceptor was measured by photoelectron spectroscopy in air (PESA), and the lowest unoccupied molecular orbital (LUMO) position were determined by adding the experimentally determined HOMO and optical bandgap energies of each molecule. The results are summarized in Figure S1 and Table S1 (Supporting Information) while Figure 1c shows the energy levels of the various materials. The HOMO level of BV was calculated to be −3.43 eV using density functional theory (DFT),^35^ which is quite close to the literature value (−3.3 eV).^30^ Critically, the HOMO of the neutral BV is significantly higher than the LUMO of the various acceptor molecules considered (i.e., IT‐4F, Y6, IT‐2Cl, EH‐IDTBR, and PC_71_BM). These favorable energetics supports the possibility of electron transfer from the BV's HOMO to the LUMO of the acceptors, leading to n‐type doping.^30^

We also performed Kelvin probe (KP) measurements to determine the work function (WF) of IT‐4F before (w/o) and after BV (0.004 wt%) doping (dash lines in Figure 1c). The WF increases by 100 meV, from −4.5 to −4.4 eV, indicating increased charge carrier density and thus successful n‐type doping of the acceptor molecule IT‐4F. Further direct experimental evidence of the n‐type doping effect were obtained via electron paramagnetic resonance (EPR) measurements performed on the pristine IT‐4F and PM6:IT‐4F blends (w/o) and their BV‐doped analogues (0.004 wt%). As shown in Figure 1d, neat IT‐4F and PM6:IT‐4F blends show small EPR signals when measured at 100 K. Addition of small amounts of BV (0.004 wt%) increases the EPR signal for both neat and blend films by approximately a factor of 15, suggesting the formation of radical anions due to electron transfer from BV to the NFA/BHJ. Based on these results, we conclude that BV acts as n‐type dopant for particular materials.

Figure 1e and Figure S3 (Supporting Information) display the absorption spectra for neat PM6, IT‐4F, and PM6:IT‐4F blend films. Addition of 0.004 wt% BV enhances the absorption coefficient (α) of IT‐4F by up to 21% (at 720 nm). In comparison, α increases by only ≈4% for PM6 (at 620 nm) after the addition of the same amount of BV. For the PM6:IT‐4F blend doped with 0.004 wt% BV, α increases by ≈7% (at 630 nm) and ≈9% (at 730 nm), when compared to neat PM6:IT‐4F layers. Increasing the BV concentration to 0.4 wt% is found to reduce α for PM6 from 8.2 × 10^4^ to 6.7 × 10^4^ cm^−1^, whilst for IT‐4F, α increases by ≈10% (Figure S3, Supporting Information). In the case of PM6:IT‐4F layers, addition of 0.4 wt% BV results in an overall decrease of α by ≈7% at 630 nm and ≈4% at 730 nm. These results show that the presence of BV affects both the electronic and optical properties of the individual materials and their blends.

Motivated by these findings, we studied the impact of BV doping on the photovoltaic properties of PM6:IT‐4F OPVs using the inverted cell architecture comprised of ITO/ZnO/BHJ/MoO_3_/Ag. First, we optimized the cells' performance by changing the BV concentration from 0.002 to 0.4 wt% (Figure S4a and Table S3, Supporting Information). **Figure** **2**a presents the current density–voltage (*J–V*) characteristics of representative cells based on undoped (w/o) and BV‐doped BHJs, while **Table** **1** summarizes the device parameters and their statistical distributions.

**Figure 2:**
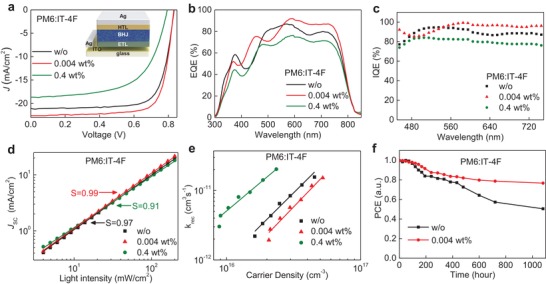
a) *J–V* characteristics of the PM6:IT‐4F BHJ cells before and after BV doping at two different weight ratios. The inset shows the schematic of the cell's architecture. b) External quantum efficiency (EQE) and c) internal quantum efficiency (IQE) spectra of the OPV cells. d) Light intensity dependence of *J*_SC_ measured for the same cells. e) Bimolecular recombination rate constant (*k*_rec_) inferred from τ and *n*, as a function of *n*. f) The 1000 h lifetime results of the OPVs based on PM6:IT‐4F with continuous testing in a dry nitrogen glove box.

**Table 1:** Summary of operating parameters of solar cells based on PM6:IT‐4F without (w/o) and with BV dopant in the BHJ (BV concentration: 0, 0.004, and 0.4 wt%)

BHJ system	BV [wt%]	BV [mol%]	*V*_OC_ [V]	*J*_SC_ (*J*_cal_)^a)^ [mA cm^−2^]	FF	PCE_max_ (PCE_avg_)^b)^ [%]	*R*_S_ [Ω cm^2^]
PM6:IT‐4F	0	0	0.83	21.1 (20.5)	0.75	13.2 (12.8)	3.4
	0.004	0.52	0.83	22.7 (22.0)	0.76	14.4 (13.9)	2.7
	0.4	51.50	0.79	18.7 (18.3)	0.61	9.1 (8.8)	6.0

^a)^ *J*_cal_ values in brackets were calculated from EQE measurements

^b)^ PCE_avg_ values in brackets represent averages from 20 devices.

Undoped devices (w/o) exhibit a maximum PCE of 13.2% with a short‐circuit current (*J*_SC_) of 21.2 mA cm^−2^, an open circuit voltage (*V*_OC_) of 0.83 V, a fill factor (FF) of 0.75, and series resistance (*R*_S_) of 3.4 Ω cm^2^. The achieved PCE is comparable to the published result for PM6:IT‐4F OPVs (13.2%) based on inverted cell architecture.^33^ Remarkably, when 0.004 wt% of BV is added into the BHJ, the cell's PCE increases sharply to 14.4%. This enhancement is accompanied by a significantly increased *J*_SC_ (22.7 mA cm^−2^), a slightly improved FF (0.76), and a reduced *R*_S_ (2.7 Ω cm^2^). Increasing the BV concentration to 0.4 wt% degrades the cell's performance yielding PCE = 9.1%, *V*_OC_ = 0.79 V, *J*_SC_ = 18.7 mA cm^−2^, and FF = 0.61, while it increases the *R*_S_ (6 Ω cm^2^). The severe degradation in *V*_OC_ observed in 0.4 wt% BV doped cells could be attributed to its higher trap concentrations created by the dopant ions.^20^

Figure 2b displays the external quantum efficiency (EQE) spectra of the PM6:IT‐4F cells. For both the neat and doped devices, the integrated current density values deduced from the EQE spectra match well the values obtained from the *J‐V* measurements within ±4%. Strikingly, the addition of 0.004 wt% BV into the BHJ results in a higher photoresponse by ≈10.5% in the spectral range 560–780 nm, as compared to the neat (w/o) device, contributing to the ≈7.6% increase in *J*_SC_. Increasing BV concentration to 0.4 wt% results in reduced photoresponse, especially in the spectral range 500–780 nm, a *J*_SC_ reduction by 11.3% and a significantly lower PCE 9.1%. The internal quantum efficiency (IQE) spectra of all cells were also measured (Figure S4b‐d, Supporting Information). The average IQE for optimally BV‐doped devices (0.004 wt%) is 94.2% in the range 450–750 nm, with a maximum value of 99% at 580 nm (Figure 2c). In comparison, the average IQEs for the neat (w/o) and 0.4 wt% BV‐doped cells were 89.2% and 79.9%, respectively. The higher average IQEs of the optimally doped cells suggest that a significantly larger portion of the absorbed photons are converted to free charge carriers which are then collected by the corresponding electrodes.

To elucidate the origin of the performance enhancement upon n‐doping, we measured the hole/electron mobilities in PM6:IT‐4F films with different layer thicknesses (100–150 nm) using the space‐charge limited current (SCLC) method (Figure S5 and Tables S4, Supporting Information). For BHJ layers doped with high BV concentration of 0.4 wt%, both the hole (μ_h_) and electron (μ_e_) mobilities increase with reducing layer thicknesses. This is not the case for neat and optimally doped (i.e., 0.004 wt%) PM6:IT‐4F layers for which the carrier mobilities remain relatively thickness‐independent. For PM6:IT‐4F layers doped with 0.004 wt% BV (thickness = 100 nm), the μ_h_ and μ_e_ appear ≈62% and 121% higher than values measured for the undoped (w/o) layers. Electron mobility appears to improve the most, possibly highlighting its key role in influencing the overall device performance. The improved balanced transport is attributed to the synergistic effects of improved charge transport due to n‐type doping and the screening of electron traps^36^ and better molecular packing,^37^ the details of which will be discussed later. Increasing the BV concentration to 0.4 wt% dramatically reduces the mobility of both carriers (μ_h_ = 3.1 × 10^−6^ cm^2^ V^−1^ s^−1^ and μ_e_ = 7.9 × 10^−6^ cm^2^ V^−1^ s^−1^). The latter is believed to be the primary reason for the degradation of the cell's overall performance (Figure 2a and Table 1).

It has previously been shown that a ratio of μ_e_/μ_h_ ≠1 can affect both the FF and *J*_SC_ of OPVs.^38^ Indeed, we find that in PM6:IT‐4F films containing a high concentration of BV (0.4 wt%), the μ_e_ is significantly higher than μ_h_, with the difference becoming more pronounced in thicker layers where the μ_e_/μ_h_ ratio is found to increase from ≈13 (100 nm) to ≈329 (150 nm). Unbalanced charge transport has been shown to lead to space charge build‐up and increased recombination,^39^ which are most likely responsible for the observed deterioration in both *J*_SC_ (18.7 mA cm^−2^) and FF (0.61). A μ_e_/μ_h_ ratio of 0.91 was obtained for BHJs with ultralow (0.004 wt%) BV concentrations yielding the highest *J*_SC_ (22.7 mA cm^−2^) and slightly enhanced FF (0.76) as compared to undoped cells.^43^, ^44^ From the analysis so far it is clear that the presence of BV affects the charge transport within the BHJ.

The impact of n‐doping on charge carrier recombination in the OPVs was also examined via light intensity (*P*_in_ in W cm^−2^) dependence *J‐V* measurements (Figure S6a‐c, Supporting Information). Earlier studies have shown that in organic BHJ cells, the *J*_SC_ usually follows the power‐law *J*_SC_ ∝ *P*_in_ *^S^*, where *S* is the power factor.^40^ A linear dependence of *J*_SC_ on *P*_in_ (*S* ≈ 1) is expected in the absence of any bimolecular recombination losses where all photogenerated carriers are successfully extracted from the device. A value of *S* < 1, on the other hand, is indicative of bimolecular recombination. Figure 2d displays the results and the corresponding *S* values. For cells doped with 0.004 wt% BV, we obtain an *S* value of 0.99, compared to 0.97 and 0.91 measured for undoped and 0.4 wt% BV‐doped cells, respectively. These results provide more evidence that small amounts of BV improve the carrier collection and reduces bimolecular recombination.^41^

Further insights into charge recombination within the device and its relation to BV doping, can be inferred from charge extraction (CE) and transient‐photovoltage (TPV) measurements.^41^, ^42^ As shown in Figure S6d (Supporting Information), the carrier density (*n*) within the cell increases upon addition of 0.004 wt% BV. Increasing the BV concentration to 0.4 wt% reduces *n* across the light intensity range investigated. This trend is in agreement with the dependence of *J*_SC_ seen in the *J*–*V* curves of Figure S6a‐c (Supporting Information). The carrier lifetime (τ) also depends on BV concentration (Figure S6e, Supporting Information). Devices with 0.004 wt% BV exhibit slightly longer τ as compared to undoped (w/o) and 0.4 wt% doped cells. Using these information the bimolecular recombination rate constants (*k*_rec_) were inferred using *k*_rec_ = 1/(λ + 1)*nτ*, where λ is the recombination order determined from the data analysis presented in Figure S6f (Supporting Information). In Figure 2e, we plot *k*_rec_ as a function of the carrier densities, for all three cells; w/o, 0.004 and 0.4 wt% BV doped. In line with the CE analysis, low BV doping concentrations (0.004 wt%) yields the lowest *k*_rec_ and a higher *n*.

To investigate the possible impact of BV‐doping on the charge generation dynamics within the BHJ layer, we performed picosecond–nanosecond transient absorption (ps–ns TA) spectroscopy measurements. To de‐convolute the contributions of singlet excitons and charges from the TA spectra, multivariate curve resolution alternating least square (MCR‐ALS) method was used.^43^, ^44^ The MCR‐ALS analysis reveals two components: singlet excitons at early delay times and charges on longer time scales. The component‐associated dynamics obtained by MCR‐ALS analysis for the undoped (w/o) and BV‐doped (0.004 and 0.4 wt%) PM6:IT‐4F systems are plotted in Figure S7 (Supporting Information). As mentioned, the exciton decay starts after 1 ps, and the charge generation follows immediately. A direct comparison of the charge carrier dynamics of the PM6:IT‐4F system clearly shows a delayed and diffusion‐limited charge generation in all three systems, where the process takes about 12, 8, and 19 ps for w/o, 0.004 and 0.4 wt% BV‐doped systems, respectively, to reach ≈50% of the maximum charge signal. This suggests that incorporation of 0.004 wt% BV improves the charge carrier generation^17^ in agreement with the aforementioned IQE measurements.

Next, we investigated the impact of BV doping on the shelf‐stability of PM6:IT‐4F solar cells. Nonencapsulated devices were stored inside a nitrogen‐filled glove box (O_2_ and H_2_O < 10 ppm) for over 1000 h and characterized via intermittent *J–V* measurements under simulated solar irradiation. Such studies provide valuable information on the evolution of the BHJ microstructure from an often kinetically frozen state reached during layer processing to a more thermodynamically stable phase over time, and/or changes occurring at the BHJ‐electrode(s) interface(s). Figure 2f shows the evolution of PCE for undoped PM6:IT‐4F (0 wt%) and n‐type doped PM6:IT‐4F:BV (0.004 wt%) BHJ cells. In both devices, the PCE decreases during the first 200 h of storage, an effect most likely attributed to morphology changes and/or diffusion of atmospheric oxidants that are still present inside the glovebox, albeit at low concentrations. Interestingly, the PCE of BV‐doped cells remains at ≈91% of its initial value as compared to that of the undoped device (≈84%). The doped cells retain their superior stability even after 1000 h of storage with the measured PCE reducing by only ≈23% as compared to pristine (w/o) devices (≈50%). Since in both types of cells, the BHJ‐electrode(s) interface(s) remain the same, any differences in the PCE degradation are most likely ascribed to changes in the microstructure of the BHJ layer.^45^ Although being preliminary, the results indicate the potential of BV‐doping for stabilizing the microstructure of the BHJ. However, a dedicated study of the impact of BV on the cell's operating stability would be required and should be the subject of a future investigation.

The influence of BV on the topography of the BHJ was examined via atomic force microscopy (AFM). **Figure** **3**a–f presents the AFM topography images for the undoped and doped BHJ layers deposited on glass substrates. Evidently, the surface of BHJ layer with 0.4 wt% BV contains large aggregates, whereas layers with 0.004 wt% BV show significantly smaller features that are comparable to those seen in the undoped (w/o) film. The different surface topographies result in differences in the surface root‐mean‐square (rms) roughness with the 0.004 wt% BV doped BHJ layer exhibiting the lowest rms (1.2 nm), followed by the w/o (1.4 nm) and 0.4 wt% BV doped (2.0 nm) films. This trend is better illustrated in the surface height histograms of Figure 3g, where the height distribution for the 0.004 wt% BV layer undergoes a clear shift toward lower heights indicating surface smoothening. The latter reflects the more compact nature of the layer (fewer voids etc.), which may contribute to the increased absorption coefficient (Figure 1e).

**Figure 3:**
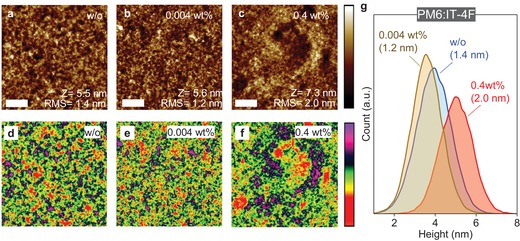
Topography AFM images of PM6:IT‐4F BHJ layers doped with; a) 0% (w/o), b) 0.004 wt% and c) 0.4 wt% BV. (scale bar: 1 µm). d–f) Topography AFM images with different color scales corresponding to (a–c). g) Surface height histograms extracted from the AFM images in (a–c).

Complementary information to AFM analysis was obtained via transmission electron microscopy (TEM) and grazing incident wide‐angle X‐ray scattering (GIWAXS) measurements (Figures S8‐S10, Supporting Information). BHJ layers incorporating a low BV concentration (0.004 wt%) exhibit similar morphologies to undoped (w/o) layers. Increasing the BV concentration to 0.4 wt%, however, results in the formation of significantly larger domains. Figure S9 (Supporting Information) displays GIWAXS data for the undoped PM6 and IT‐4F films, while Figure S10 (Supporting Information) presents data measured for the pristine and BV‐doped BHJ layers. In the 2D diffraction images for the undoped (w/o) BHJ (Figure S10a, Supporting Information), the (100) peak located at 0.30 A^−1^ is composed of PM6 and IT‐4F lamellar packing features seen in Figure S9 (Supporting Information). The π–π stacking at 1.77 A^−1^ is also composed of both material features since the π–π stacking of PM6 and IT‐4F are located at 1.72 and 1.80 A^−1^, respectively. No obvious changes, i.e., appearance or vanishing of new peaks, are observed upon doping (Figure S10b, Supporting Information), meaning that the presence of BV does not affect the molecular orientation. The crystalline stacking order of the systems was also studied by comparing the crystal intensity in the various samples of similar thickness (Figure S10c,d, Supporting Information). Interestingly, the degree of microcrystallinity in the PM6:IT‐4F film reduces significantly in both out‐of‐plane and in‐plane direction upon addition of 0.4 wt% BV, suggesting higher than optimal dopant concentrations weaken both the lamellar packing and π–π stacking order. On the other hand, lowering the BV concentration to 0.004 wt% enhances slightly the crystal intensity in out‐of‐plane, indicating that ultralow concentrations of BV somewhat strengthens the π–π stacking but with negligible effect on the molecular orientation. The GIWAXS results support the enhanced absorption characteristics of the PM6:IT‐4F doped with 0.004 wt% BV (Figure 1e) and possibly the different charge generation dynamics (Figure S7, Supporting Information) as well as the improved shelf stability of PM6:IT‐4F:BV (0.004 wt%) cells. The data are also in agreement with the AFM results, which suggest the formation of more compact BHJ layers upon optimal BV‐doping.

Finally, we investigate the broader applicability of BV to other promising BHJ systems. Five additional blends namely; i) PM6:Y6:PC_71_BM,^24^ ii) PM6:Y6,^34^ iii) PM6:IT‐2Cl,^33^ iv) poly[[4,8‐bis[5‐(2‐ethylhexyl)thiophene‐2‐yl]benzo[1,2‐b:4,5‐b0]dithiophene‐2,6‐diyl][3‐fluoro‐2‐[(2‐ethylhexyl)carbonyl]‐thieno[3,4‐b]thiophenediyl]] (PTB7‐Th):EH‐IDTBR,^20^ and v) PTB7‐Th:PC_71_BM^46^ (Figures S11‐S15 and Tables S5‐S9, Supporting Information) were investigated. The HOMO and LUMO energies of the materials are shown in Figure 1c. **Figure** **4**a presents the measured PCE for each BHJ system without (w/o) and with BV (0.004 and 0.4 wt%) while **Table** **2** summarizes the cells' parameters. Here we note that the optimal wt% of BV for PTB7‐Th:EH‐IDTBR and PTB7‐Th:PC_71_BM blends was 0.002 wt%. Evidently, the introduction of BV, at optimal concentrations, in all BHJ systems studied enhance the cells' PCE when compared to pristine (undoped) devices. Increasing the BV wt% beyond the optimal concentration results in performance deterioration as evident by the plummeting PCE. Analysis of the device characteristics shows that in the case of the optimally doped (0.004 wt%) cells, the increased PCE is attributed to the lower *R*_S_, the improved *J*_SC_, and the generally higher photoresponse, in agreement with the findings for the PM6:IT‐4F cells. OPVs based on PM6:Y6 and PM6:Y6:PC_71_BM yield the highest performance with maximum PCEs of 16.0% and 17.1%, respectively (Figure 4b). The *J*‐*V* data for 20 different but simultaneously fabricated OPV cells based on PM6:Y6:PC_71_BM BHJs doped with 0.004 wt% of BV, are presented in Figure S16 (Supporting Information), while a summary of cell parameters is given in Table S10 (Supporting Information). In Figure 4c, we summarize the PCE values reported to date for OPVs based on molecularly doped BHJs.^17^, ^18^, ^20^, ^22^, ^47^, ^48^ Among these studies Xu et al.,^22^ is the only one to describe the impact of n‐type doping in all‐polymer OPVs with the remaining reporting the use of p‐type dopants.

**Figure 4:**
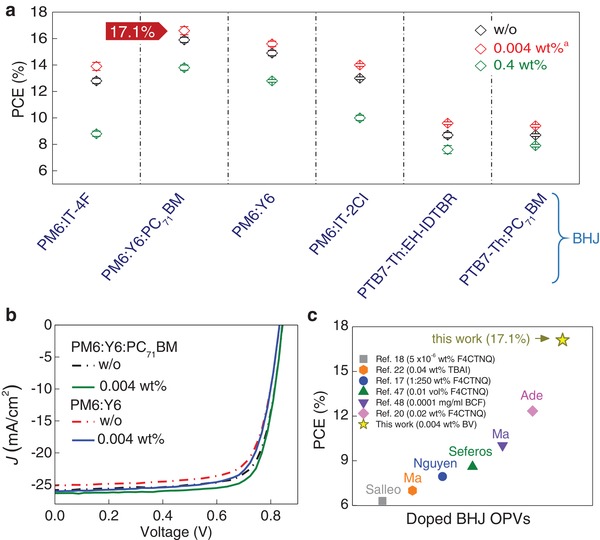
a) Summary of the PCE values of different BHJ systems doped with different weight ratios of BV.^a^ The optimal BV concentration for the PTB7‐Th:EH‐IDTBR and PTB7‐Th:PC_71_BM systems was 0.002 wt%. b) *J–V* curves of OPVs based on PM6:Y6 and PM6:Y6:PC_71_BM systems without doping (w/o) and with 0.004 wt% BV. c) A comparison of reported PCE values for OPVs based on doped BHJ layers, and corresponding references and dopants used (TBAI is n‐type dopant and others are p‐type dopant).

**Table 2:** Summary of operating parameters of solar cells based on five different BHJ systems without (w/o) and with two BV concentrations

BHJ system	BV [wt%]	BV [mol%]	*V*_OC_ [V]	*J*_SC_ (*J*_cal_)^a)^ [mA cm^−2^]	FF	PCE_max_ (PCE_avg_)^b)^ [%]	*R*_S_ [Ω cm^2^]
PM6:Y6: PC_71_BM	0	0	0.84	25.7 (25.5)	0.75	16.3 (15.9)	3.3
	0.004	0.54	0.84	26.3 (26.0)	0.77	17.1 (16.6)	2.5
	0.4	53.76	0.83	25.3 (24.6)	0.67	14.2 (13.8)	4.5
PM6:Y6	0	0	0.83	25.1 (24.8)	0.73	15.3 (14.9)	2.9
	0.004	0.54	0.83	26.0 (25.5)	0.74	16.0 (15.6)	2.8
	0.4	53.67	0.82	24.3 (24.2)	0.66	13.1 (12.8)	4.0
PM6:IT‐2Cl	0	0	0.89	20.8 (20.4)	0.72	13.3 (13.0)	4.1
	0.004	0.52	0.89	22.0 (21.7)	0.73	14.3 (14.0)	3.3
	0.4	51.52	0.86	19.1 (19.0)	0.64	10.4 (10)	6.0
PTB7‐Th: EH‐IDTBR	0	0	1.02	15.8 (15.9)	0.57	9.1 (8.7)	11.1
	0.002	0.19	1.02	16.4 (15.5)	0.60	9.9 (9.6)	10.3
	0.4	38.78	1.01	15.6 (15.5)	0.52	8.1 (7.6)	11.3
PTB7‐Th: PC_71_BM	0	0	0.80	17.5 (17.0)	0.65	9.0 (8.7)	5.4
	0.002	0.23	0.80	18.2 (17.9)	0.66	9.6 (9.4)	4.7
	0.4	46.13	0.78	16.9 (16.4)	0.61	8.0 (7.9)	6.2

^a)^ *J*_cal_ values in brackets were calculated from EQE measurements

^b)^ PCE_avg_ values in brackets represent averages from 20 devices.

In conclusion, we have shown that addition of small amounts of the molecular n‐type dopant BV directly into the BHJ layer of best‐in‐class organic solar cells, leads to consistent performance improvements. The influence of BV was shown to be twofold, first acting as an n‐type dopant which improves the electron transport within the BHJ, and second as a microstructure modifier. These synergistic effects result in more balanced bipolar transport and stronger light absorption within the BHJ. In the case of solar cells based on PM6:IT‐4F blends, addition of only 0.004 wt% BV increases the PCE from 13.2% to a maximum value of 14.4%. The enhanced PCE is the direct result of the higher photoresponse in the longer wavelength range leading to a 7.6% increase of *J*_SC_. Increasing the BV concentration to 0.4 wt% was found to rapidly degrade the cell's performance due to the unbalanced carrier mobilities. Analysis of the BHJs microstructure shows that the presence of BV in optimal concentrations strengthens the π–π stacking but has no obvious effect on the molecular orientation. Increasing the BV concentration beyond optimal levels weakened both the lamellar packing and π–π stacking order within the BHJ. Overall, the combination of these BV‐induced effects leads to consistently improved charge generation, faster charge transport, improved charge extraction efficiency, and lower carrier recombination losses in a wide range of state‐of‐the‐art organic BHJ systems. The broad versatility of BV is culminated with its application in ternary OPVs cells based on PM6:Y6:PC_71_BM for which the PCE is shown to increase from 16.3% (undoped) to 17.1% (0.004 wt% BV). The universality of BV combined with the remarkably high PCE values obtained, makes this simple n‐type dopant strategy promising for application in high‐performance OPVs. It also highlights electron transport as a limiting factor in state‐of‐the‐art NFA‐based OPVs.

## Experimental Section

##### Dopant Solution Preparation

The BV solution was prepared according to the previously published procedures.^30^ Briefly, 1,1′‐dibenzyl‐4,4′‐bipyridinium dichloride hydrate (51 mg/0.12 mmol) was dissolved in distilled water (4.5 mL). Toluene (9.0 mL) was slowly dropped on the aqueous layer, and sodium borohydride (97 mg/2.5 mmol) was added into the bilayer system. The colorless aqueous layer immediately became deep violet in color with the generation of hydrogen gas. After 12 h, the aqueous layer became colorless while the top toluene layer was yellow. The toluene layer was separated and removed under vacuum for solvent exchange with chlorobenzene (CB) or chloroform (CF).

##### Solar Cell Fabrication

PM6 (51.2 kDa), IT‐4F, Y6, IT‐2Cl, and PC_71_BM were purchased from Solarmer Materials Inc. PTB7‐Th (57.5 kDa) was purchased from 1‐Materials Inc. EH‐IDTBR was synthesized in‐house using previously published procedures.^49^ The ZnO precursor solution was prepared by dissolving 200 mg of zinc acetate dihydrate in 2 mL of 2‐methoxyethanol and 60 µL of 2‐aminoethanol. For the BHJ solutions, the PM6:IT‐4F and PM6:IT‐2Cl (D:A = 1:1, 20 mg mL^−1^ in total) materials were dissolved in CB with the 1,8‐diiodooctane (DIO) (0.5%, v/v). The PM6:Y6:PC_71_BM (D:A = 1:1:0.2, 15 mg mL^−1^ in total) and PM6:Y6 (D:A = 1:1.2, 15 mg mL^−1^ in total) was dissolved in CF with chloronaphthalene (CN) (0.5%, v/v). The PTB7‐Th:EH‐IDTBR (D:A = 1:2, 30 mg mL^−1^ in total) was dissolved in CB. The PTB7‐Th:PC_71_BM (D:A = 1:1.5, 25 mg mL^−1^ in total) was dissolved in CB with the DIO (3%, v/v). Addition of the desired concentration (0, 0.01, 0.05, and 0.1 mg mL^−1^) of BV (dissolved in CB or CF) was performed via physical blending of the required amount of the dopant solution directly into the BHJ solution and stirred for 30 min. Indium tin oxide (ITO) coated glass substrates (Kintec Company, 10 Ω sq.^−1^) were cleaned by sequential ultrasonication in dilute Extran 300 detergent solution, deionized water, acetone, and isopropyl alcohol for 10 min each. The substrates were then subjected to a UV–ozone treatment step for 20 min. Next, ZnO precursor solution was spin‐coated onto the substrates and then dried on a hot plate at 200 °C for 0.5 h. The samples were then transferred into a dry nitrogen glove box (≈10 ppm O_2_). The active solutions were then spun to obtain an active‐layer thickness in the narrow range of 95–105 nm (For PM6:Y6:PC_71_BM and PM6:Y6, the thickness is around 150–160 nm). Finally, the samples were placed in a thermal evaporator and 7 nm of MoO_3_ and 100 nm of aluminum were then thermally evaporated at 5 × 10^−7^ mbar through a 0.1 cm^2^ pixel area shadow mask. For stability tests, as‐fabricated and un‐encapsulated devices were stored inside a nitrogen glove box (O_2_ and H_2_O < 10 ppm) for over 1000 h and characterized via intermittent *J–V* measurements under simulated solar irradiation.

##### Device Characterization

UV–vis spectra were recorded on a Cary 5000 instrument in single beam mode in 1 cm quartz cuvettes. *J*–*V* measurements of solar cells were performed in a N_2_ filled glove box using a Keithley 2400 source meter and an Oriel Sol3A Class AAA solar simulator calibrated to 1 sun, AM1.5G, with a KG‐5 silicon reference cell certified by Newport. EQE was characterized using an EQE system (PV measurement Inc.). Measurements were performed at zero bias by illuminating the device with monochromatic light supplied from a Xenon arc lamp in combination with a dual‐grating monochromator. The number of incident photons on the sample was calculated for each wavelength by using a silicon photodiode calibrated by The National Institute of Standards and Technology (NIST). The internal quantum efficiency of each OPV cell was calculated using: IQE(%) = EQE(%)/(100% − Reflectance(%) − Parasitic Absorption(%)). The reflectance spectra were collected with the integrating sphere using the same EQE system while the parasitic absorption spectra were obtained from transfer matrix modelling. A Bruker AFM was used to image the surface of the various layers in tapping mode.

##### Electron Microscopy and X‐Ray Diffraction Measurements

TEM studies analyses were performed using a FEI Titan 80‐300 TEM equipped with an electron monochromator and a Gatan Imaging Filter (GIF) Quantum 966. GIWAXS characterization of active layer was performed at beamline 7.3.3, Advanced Light Source (ALS), Lawrence Berkeley National Lab (LBNL). X‐ray energy was 10 keV and operated in top off mode. The scattering intensity was recorded on a 2D image plate (Pilatus 2M). The GIWAXS experiment was done in a closed chamber purged with Helium gas to suppress air scattering. The chamber was sealed using kapton films, which give rise to blank cell scattering features.

##### EPR Measurement

All EPR spectra were recorded using X‐band continuous wave Bruker EMX PLUS spectrometer (BrukerBioSpin, Rheinstetten, Germany) equipped with standard resonator for high sensitivity continuous‐wave electron paramagnetic resonance and operating at (9.384688) GHz. The low temperature (100 K) spectra were measured using a liquid helium setup with 25 dB microwave attenuation with 5 GHz modulation amplitude and 100 kHz modulation frequency.

##### Density Functional Theory Calculation

The results were obtained with density functional theory calculations using the NWChem code,[qv: 50a] the hybrid B3LYP exchange‐correlation functional[qv: 50b] and the DZVP DFT Orbital basis.[qv: 50c] The HOMO and LUMO orbitals were rendered with VESTA.^34^

##### Light‐Intensity Dependence Measurements

Light‐intensity dependence measurements were performed with PAIOS instrumentation (Fluxim) (steady‐state and transient modes). Transient photo‐voltage measurements monitor the photovoltage decay upon a small optical perturbation during various constant light‐intensity biases and at open‐circuit bias condition. Variable light‐intensity biases lead to a range of measured *V*_OC_ values that were used for the analysis. During the measurements a small optical perturbation (<3% of the *V*_OC_, so that ∆*V*_OC_ << *V*_OC_) is applied. The photovoltage decay kinetics of all devices follow a mono‐exponential decay: *δV* = *A* exp(‐*t*/τ), where *t* is the time and τ is the charge carrier lifetime. The “charge extraction” technique was used to measure the charge carrier density *n* under open‐circuit voltage condition. The device is illuminated and kept in open‐circuit mode. After light turn‐off, the voltage is switched to zero or taken to short‐circuit condition to extract the charges. To obtain the number of extracted charges, the current is integrated. The carrier lifetimes follow a power law relationship with charge density: τ = τ_0_ *n* ^−^ *^λ^*. The bimolecular recombination constant *k*_rec_ were then inferred from the carrier lifetimes and densities according to *k*_rec_ = 1/(λ + 1)/*nτ* ^2^, where λ is the recombination order.

##### TA Spectroscopy

Transient absorption (TA) spectroscopy was carried out using a home‐built pump‐probe setup. The output of titanium:sapphire amplifier (Coherent LEGEND DUO, 4.5 mJ, 3 kHz, 100 fs) was split into three beams (2, 1, and 1.5 mJ). Two of them were used to separately pump two optical parametric amplifiers (OPA) (Light Conversion TOPAS Prime). The TOPAS 1 generates pump pulses to excite the sample, while the TOPAS 2 generates signal (1300 nm) and idler (2000 nm) only. TOPAS 1 was used for producing pump pulses while the probe pathway length to the sample was kept constant at ≈5 m between the output of the TOPAS 1 and the sample. The pump pathway length was varied between 5.12 and 2.6 m with a broadband retroreflector mounted on automated mechanical delay stage (Newport linear stage IMS600CCHA controlled by a Newport XPS motion controller), thereby generating delays between pump and probe from −400 ps to 8 ns. For measuring TA of whole visible range, 1300 nm (signal) of TOPAS 2 was used to produce white‐light super continuum from 350 to 1100 nm. The transmitted fraction of the white light was guided to a custom‐made prism spectrograph (Entwicklungsbüro Stresing) where it was dispersed by a prism onto a 512 pixel NMOS linear image sensor (hamamatsu S8381‐512). The probe pulse repetition rate was 3 kHz, while the excitation pulses were mechanically chopped to 1.5 kHz (100 fs to 8 ns delays) while the detector array was read out at 3 kHz. Adjacent diode readings corresponding to the transmission of the sample after excitation and in the absence of an excitation pulse were used to calculate Δ*T*/*T*. Measurements were averaged over several thousand shots to obtain a good signal‐to‐noise ratio. The chirp induced by the transmissive optics was corrected with a home‐built Matlab code. The delay at which pump and probe arrive simultaneously on the sample (i.e., zero time) was determined from the point of maximum positive slope of the TA signal rise for each wavelength.

## Conflict of Interest

The authors declare no conflict of interest.

## Supporting information

Supporting Information
