# Supplementary material for: 17.1% Efficient Single‐Junction Organic Solar Cells Enabled by n‐Type Doping of the Bulk‐Heterojunction
Source: Adv Sci (Weinh). 2020 Feb 13;7(7):1903419. doi: 10.1002/advs.201903419 (PMC7141031; doi:10.1002/advs.201903419)
Supplement: Supplementary file 1 — Supporting Information [file ADVS-7-1903419-s001.pdf]

Copyright WILEY-VCH Verlag GmbH & Co. KGaA, 69469 Weinheim, Germany, 2020.

ADVANCED SCIENCE
Open Access

Supporting Information

for *Adv. Sci.,* DOI: 10.1002/advs.201903419

17.1% Efficient Single-Junction Organic Solar Cells Enabled by n-Type Doping of the Bulk-Heterojunction

*Yuanbao Lin, Yuliar Firdaus, Mohamad Insan Nugraha, Feng Liu, Safakath Karuthedath, Abdul-Hamid Emwas, Weimin Zhang, Akmaral Seitkhan, Marios Neophytou, Hendrik Faber, Emre Yengel, Iain McCulloch, Leonidas Tsetseris, Frédéric Laquai, and Thomas D. Anthopoulos**

Copyright WILEY-VCH Verlag GmbH & Co. KGaA, 69469 Weinheim, Germany, 2020.

Supporting Information

17.1% Efficient Single-Junction Organic Solar Cells Enabled by *n*-Type Doping of the Bulk-Heterojunction

Yuanbao Lin, Yuliar Firdaus, Mohamad Insan Nugraha, Feng Liu, Safakath Karuthedath, Abdul-Hamid Emwas, Weimin Zhang, Akmaral Seitkhan, Marios Neophytou, Hendrik Faber, Emre Yengel, Iain McCulloch, Leonidas Tsetseris, Frédéric Laquai, Thomas D. Anthopoulos*

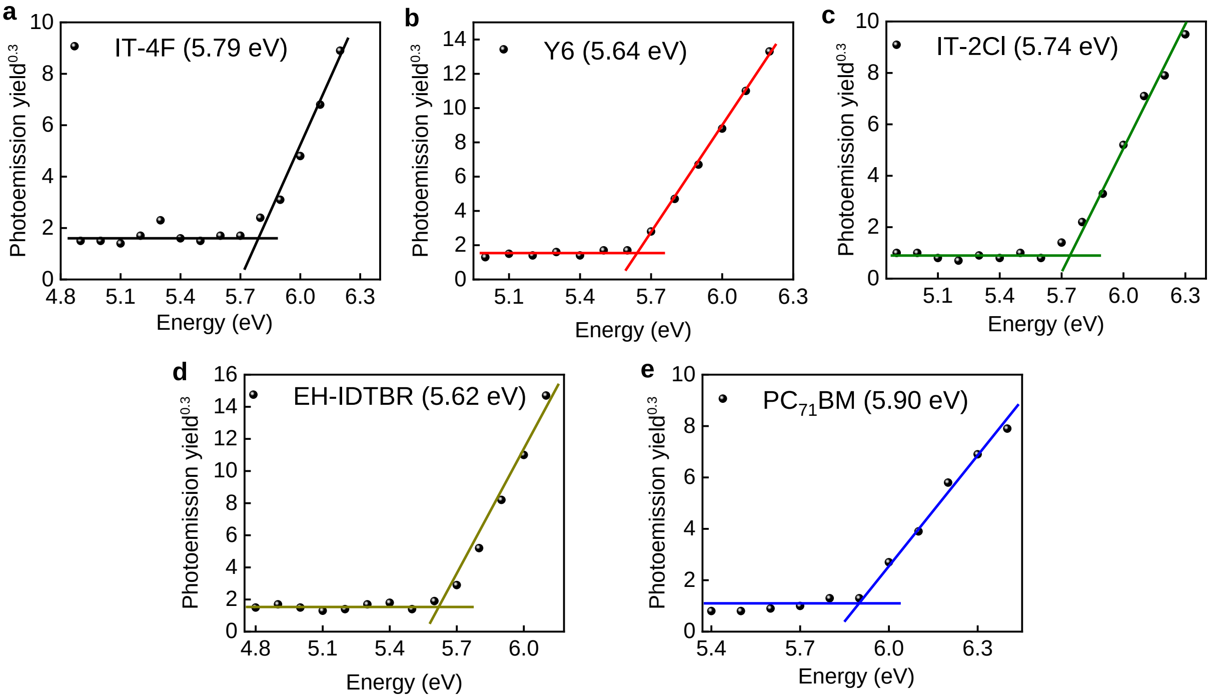

Figure S1. PESA measurement of the electron-acceptor materials a) IT-4F, b) Y6, c) IT-2Cl, d) EH-IDTBR, and e) $PC_{71}BM$ investigated in this study.

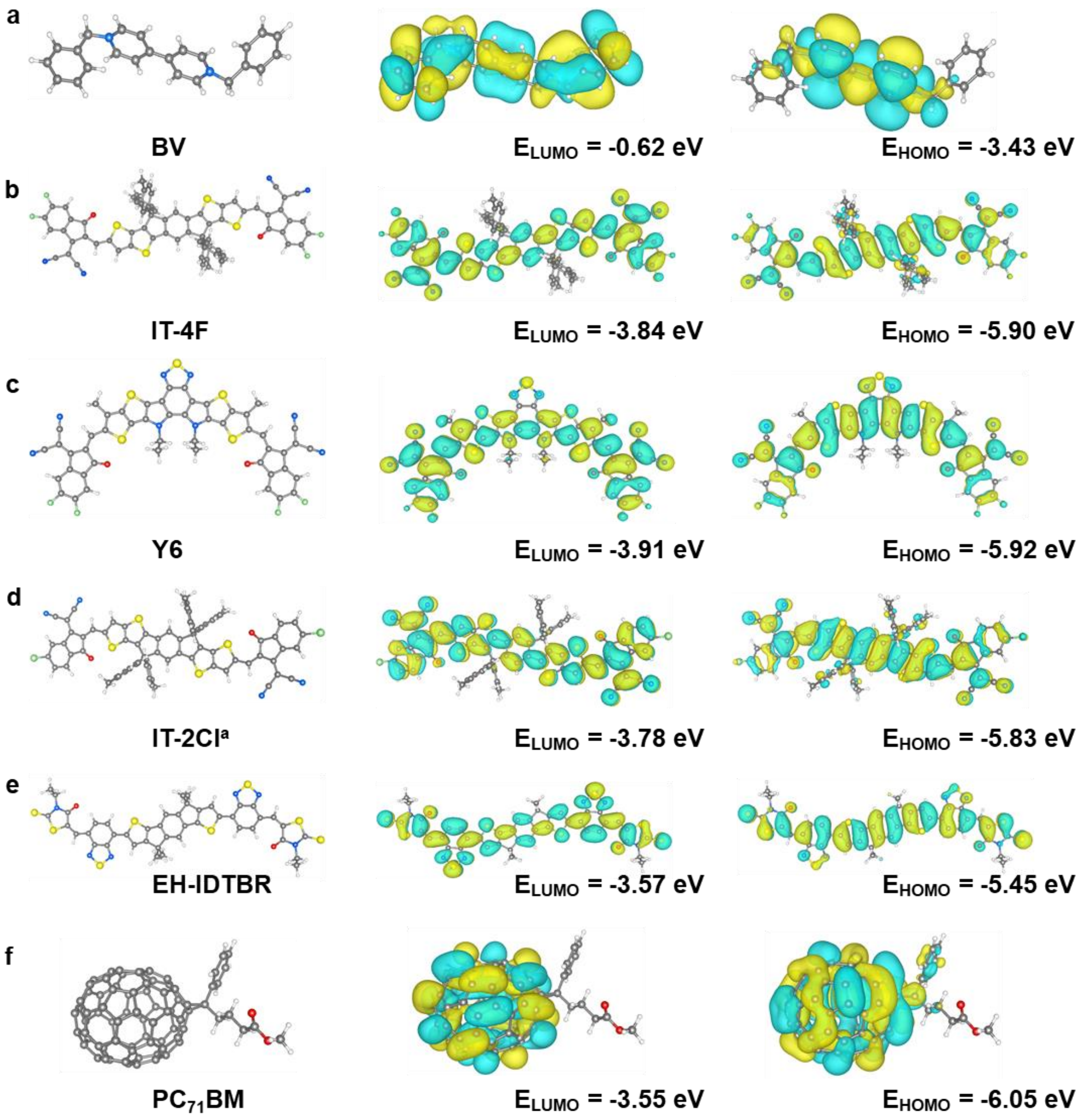

Figure S2. a)-f) Molecular structure of the *n*-type dopant BV and the various electron-acceptors investigated in this study, and their highest occupied molecular orbitals (HOMO) and lowest unoccupied molecular orbitals (LUMO) calculated via DFT (C: gray, H: white, S: yellow, O: red, N: blue, F: light green spheres). [a]For IT-2Cl, light green spheres represent Cl atoms.

Table S1. LUMO and HOMO of various materials evaluated by PESA and DFT calculations.

Material	PESA		DFT		Ref.
	LUMO [eV][a]	HOMO [eV]	LUMO [eV]	HOMO [eV]	HOMO [eV]
BV	-	-	-0.62	-3.43	-3.28[1]
IT-4F	-4.24	-5.79	-3.84	-5.90	-
Y6	-4.29	-5.64	-3.91	-5.92	-
IT-2Cl	-4.17	-5.72	-3.78	-5.83	-
EH-IDTBR	-3.92	-5.59	-3.57	-5.45	-
$PC_{71}BM$	-4.15	-5.90	-3.55	-6.05	-

[a] LUMO values were calculated by adding HOMO with optical band gaps.

Table S2. Work function of PM6 and IT-4F were measured from Kelvin Probe.

Solid surface		Work function[eV]
IT-4F	0 wt%	-4.5
	0.004 wt%	-4.4

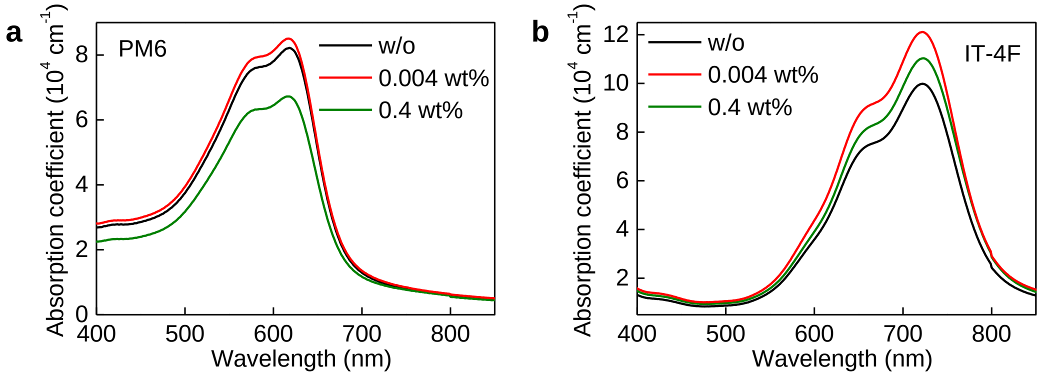

Figure S3. Absorption profiles of neat (0%) PM6 and IT-4F films and doped with 0.004 and 0.4 wt% BV.

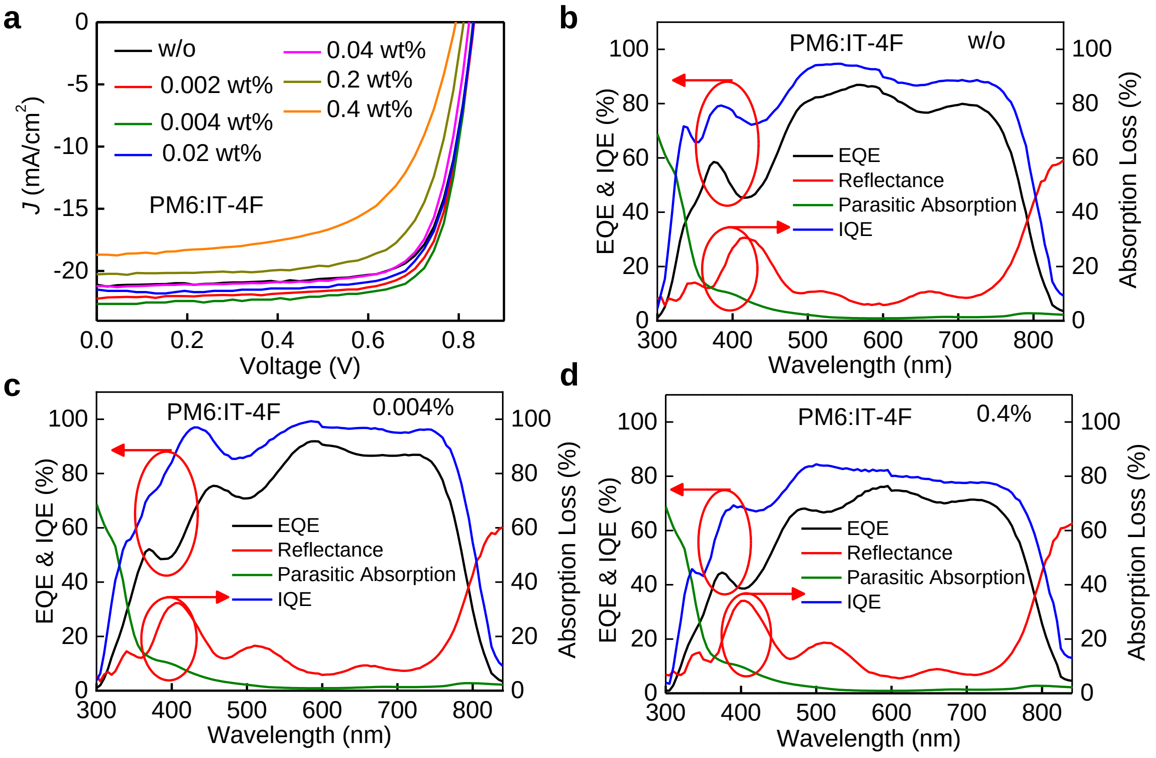

Figure S4. a) *J-V* curves of OPV cells based on PM6:IT-4F doped with different wt% of BV. EQE, IQE, reflectance, and parasitic absorption spectra of OPV cells based on PM6:IT-4F doped with b) 0 wt%, c) 0.004 wt% and d) 0.4 wt% BV.

Table S3. Summary of photovoltaic operating parameters for PM6:IT-4F OPVs doped with different wt% of BV, measured under AM 1.5G illumination (100 mW/cm^2).

BV [wt%]	BV [mol%]	V_{OC} [V]	J_{SC} (J_{cal})[a] [mA/cm^2]	FF	PCE_{max} (PCE_{avg})[b] [%]
0	0	0.83	21.1 (20.5)	0.75	13.2 (12.8±0.2)
0.002	0.26	0.83	22.2	0.75	13.9 (13.3±0.4)
0.004	0.52	0.83	22.7 (22.0)	0.76	14.4 (13.9±0.3)
0.02	2.58	0.83	21.5	0.76	13.6 (13.1±0.3)
0.04	5.15	0.82	21.2	0.75	13.1 (12.7±0.3)
0.2	25.75	0.81	20.3	0.71	11.7 (11.1±0.4)
0.4	51.50	0.79	18.7 (18.3)	0.61	9.1 (8.8±0.2)

[a] J_{cal} values in brackets were calculated from EQE measurements.
[b] PCE_{avg} values in brackets represent averages from 20 devices.

S1. SCLC analysis

The carrier mobilities of the PM6:IT-4F were determined by fitting the dark current to the space-charge-limited current (SCLC) model. The device structures were glass/ITO/PEDOT:PSS/BHJ/MoO_3/Ag (Hole-only devices) and glass/ITO/ZnO/BHJ/PFN-Br/Ag (Electron-only devices). The electric-field dependent SCLC mobility was estimated using Equation S1:

$$J\ (\mathrm{V}) = \frac{9}{8}\varepsilon_0\varepsilon_r\mu_0 exp\left(0.89\beta\sqrt{\frac{V-V_{bi}}{L}}\right)\frac{(V-V_{bi})^2}{L^3} \quad \text{(S1)}$$

Term definition	Symbol	Units
zero-field mobility	μ_0	$cm^2\ V^{-1}\ s^{-1}$
film thickness	L	cm
dark current density	J	$mA\ cm^{-2}$
voltage	V	V
vacuum permittivity	ε_0 (88.54 × 10^{-12})	$mA\ s\ V^{-1}\ cm^{-1}$
dielectric constant	ε_r (3)	
field activation factor	β	$cm^{1/2}\ V^{-1/2}$

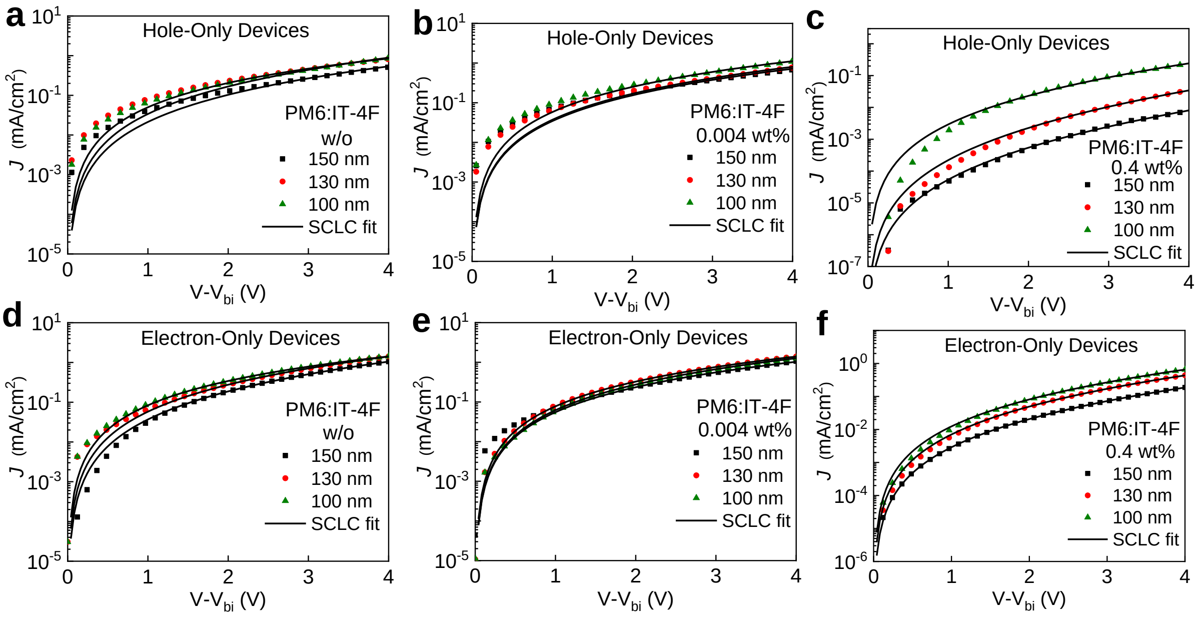

Figure S5. Experimental dark current densities as a function of voltage for a)-c) hole-only devices and d)-f) electron-only devices made with blend films of PM6:IT-4F doped with 0 wt% (w/o), 0.004 wt% 0.4 wt% BV. The experimental data were fitted using the single carrier SCLC model as described above.

Table S4. Zero-field hole and electron mobilities of PM6:IT-4F doped with 0 wt% (w/o), 0.4 wt% and 0.004 wt% BV and for different film thicknesses.

BV [wt%]	Film Thickness [nm]	μ_h [cm^2 V^{-1}s^{-1}]	μ_e [cm^2 V^{-1}s^{-1}]	μ_e/μ_h
w/o	150	(2.2±0.2) ×10^{-4}	(1.3±0.2) ×10^{-4}	0.59
	130	(2.3±0.2) ×10^{-4}	(1.4±0.5) ×10^{-4}	0.61
	100	(2.1±0.5) ×10^{-4}	(1.4±0.7) ×10^{-4}	0.66
0.004	150	(2.5±0.6) ×10^{-4}	(1.9±0.1) ×10^{-4}	0.76
	130	(3.1±0.8) ×10^{-4}	(2.7±0.2) ×10^{-4}	0.87
	100	(3.4±0.5) ×10^{-4}	(3.1±0.7) ×10^{-4}	0.91
0.4	150	(1.7±0.2) ×10^{-8}	(5.6±1.6) ×10^{-6}	329.41
	130	(1.6±0.8) ×10^{-7}	(7.9±1.1) ×10^{-6}	49.38
	100	(1.1±0.7) ×10^{-6}	(1.4±0.3) ×10^{-5}	12.73

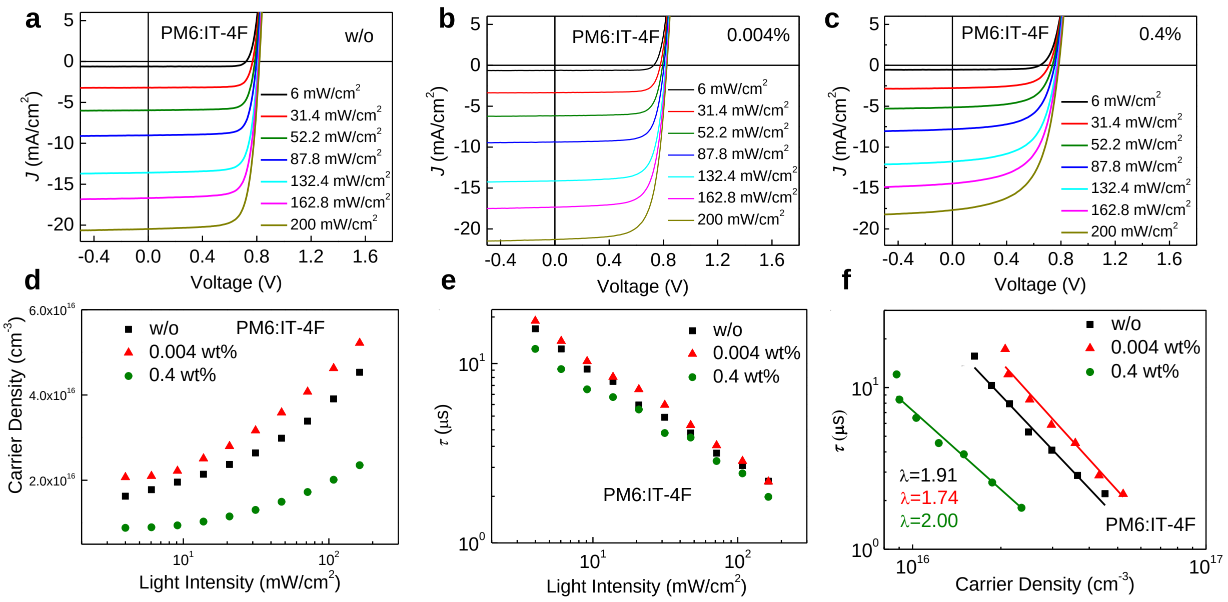

Figure S6. *J-V* curves *vs.* light intensity for OPV cells doped with a) 0 wt%, b) 0.004 wt%, and c) 0.4 wt%. d) Charge density *vs.* light intensity, and e) charge carrier lifetime (τ). f) Charge carrier lifetime (τ) *vs.* charge density.

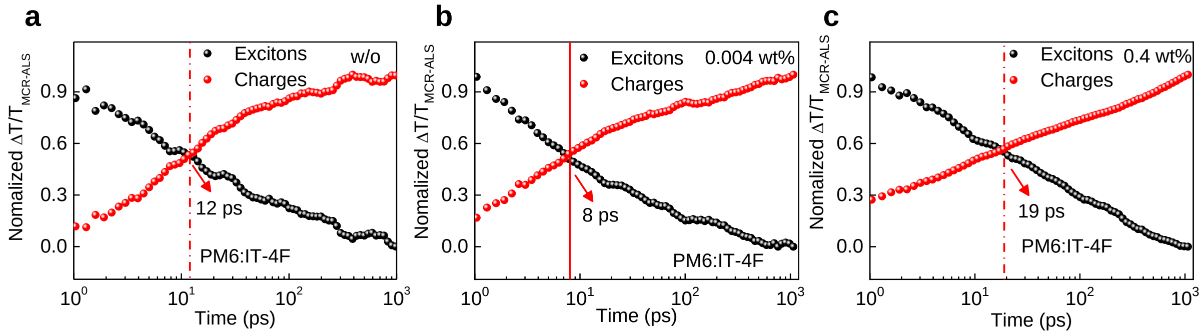

Figure S7. Component dynamics as extracted by the MCR-ALS analysis for excitons and charge carriers at different fluences for: a) 0%, b) 0.004 wt% and c) 0.4 wt% BV.

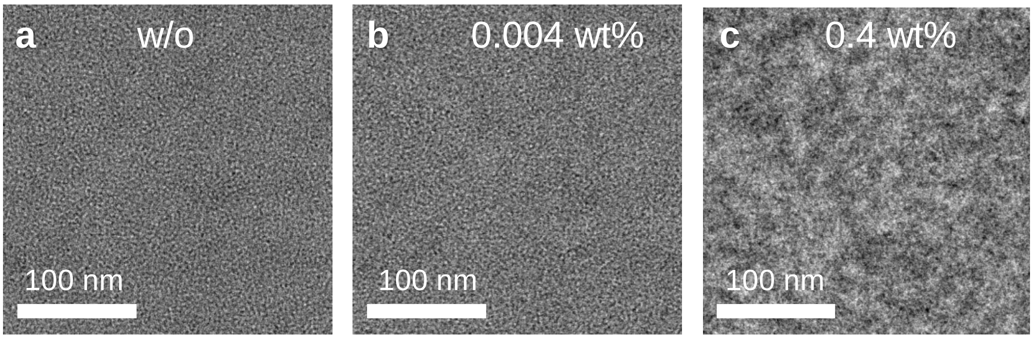

Figure S8. Transmission electron microscopy (TEM) images of PM6:IT-4F (1:1, w/w) doped with: a) 0 wt% (w/o), b) 0.004 wt% and c) 0.4 wt% of BV.

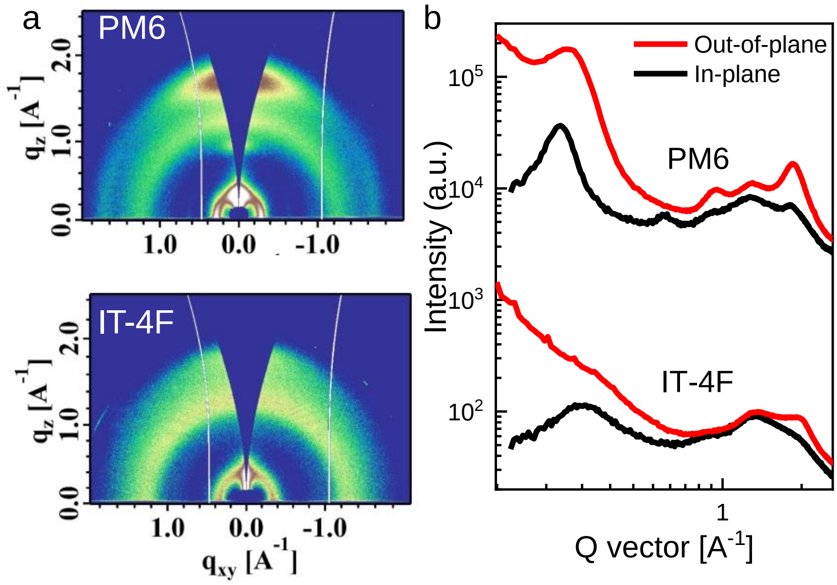

Figure S9. a) 2-D GIWAXS images of the neat PM6 and neat IT-4F films. b) In-plane and out-of-plane line cut profiles.

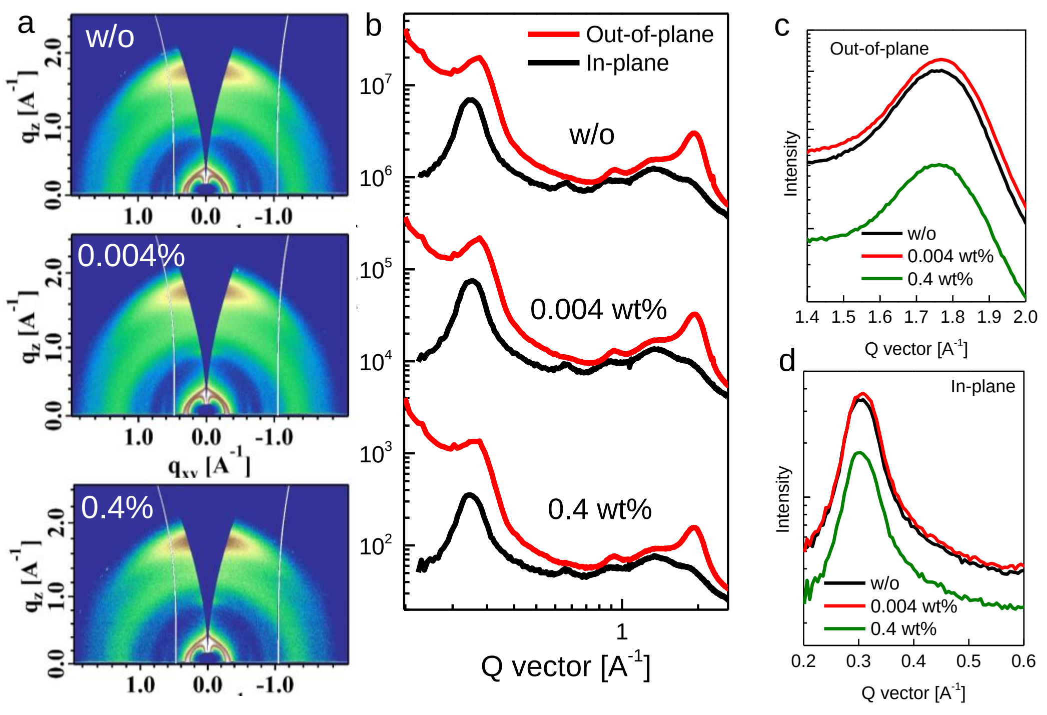

Figure S10. a) 2-D GIWAXS images of the PM6:IT-4F films doped with 0 wt%, 0.004 wt% and 0.4 wt% BV. b)-d) In-plane and out-of-plane line cut profiles.

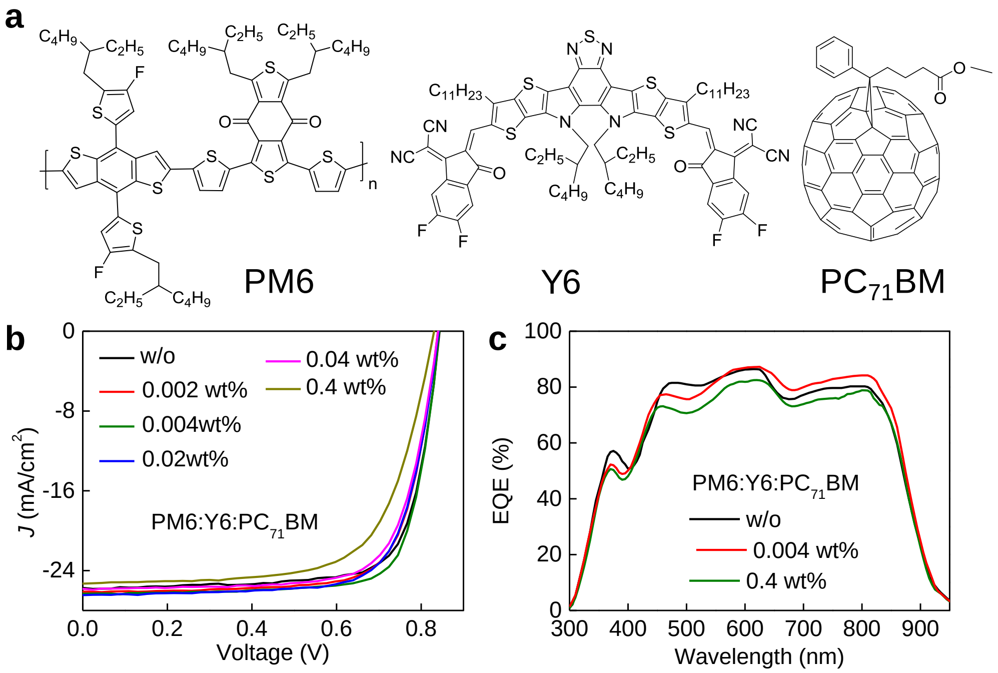

Figure S11. a) Chemical structures of PM6, Y6 and $PC_{71}BM$. b) *J-V* curves and c) EQE curves of OPV cells based on PM6:Y6:$PC_{71}BM$ doped with different wt% of BV.

Table S5. Summary of photovoltaic operating parameters for PM6:Y6:$PC_{71}BM$ OPVs doped with different wt% of BV, measured under illumination of AM 1.5G (100 mW/cm^2).

BV [wt%]	BV [mol%]	V_{OC} [V]	J_{SC} (J_{cal})[a] [mA/cm^2]	FF	PCE_{max} (PCE_{avg})[b] [%]
0	0	0.84	25.7 (25.5)	0.75	16.3 (15.9±0.2)
0.002	0.27	0.84	26.1	0.74	16.3 (16.0±0.1)
0.004	0.54	0.84	26.3 (26.0)	0.77	17.1 (16.6±0.3)
0.02	2.69	0.84	26.5	0.73	16.3 (16.0±0.1)
0.04	5.38	0.84	25.8	0.73	15.9 (15.5±0.2)
0.4	53.76	0.83	25.3 (24.6)	0.67	14.2 (13.8±0.2)

[a] J_{cal} values in brackets were calculated from EQE measurements.
[b] PCE_{avg} values in brackets represent averages from 20 devices.

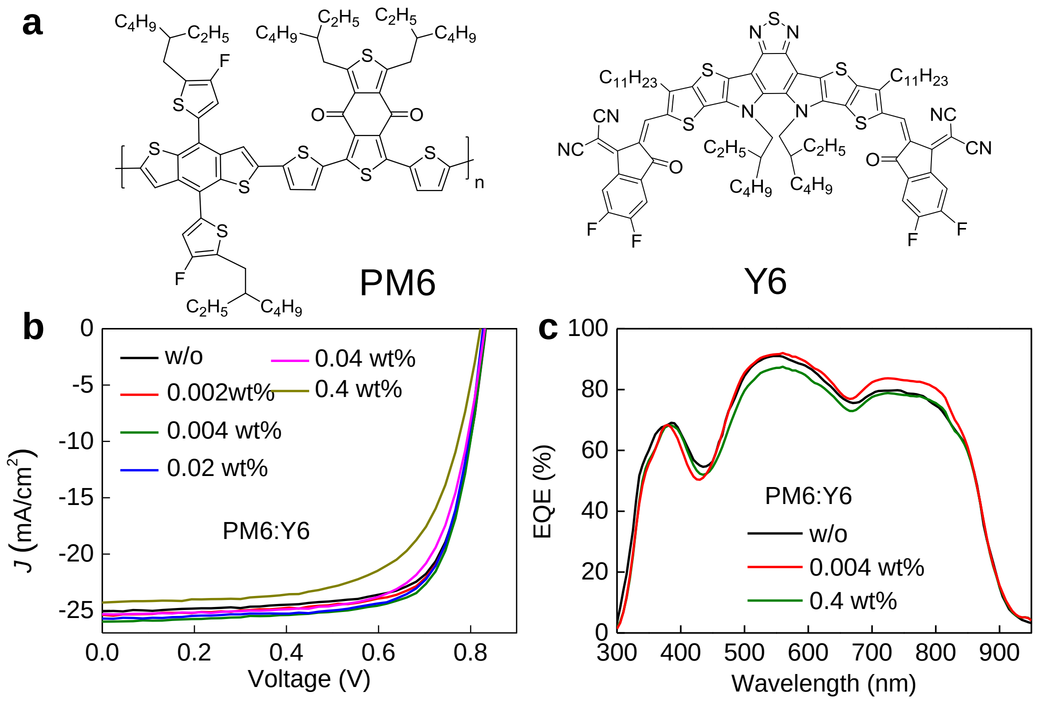

Figure S12. a) Chemical structures of PM6 and Y6. b) *J*-*V* curves and c) EQE curves of OPV cells based on PM6:Y6 doped with different weight ratios of BV.

Table S6. Summary of photovoltaic operating parameters for PM6:Y6 OPVs doped with different wt% of BV. All cells were measured under illumination of AM 1.5G (100 mW/cm^2).

BV [wt%]	BV [mol%]	V_{OC} [V]	J_{SC} (J_{cal})[a] [mA/cm^2]	FF	PCE_{max} (PCE_{avg})[b] [%]
0	0	0.83	25.1 (24.8)[a]	0.73	15.3 (14.9±0.2)[b]
0.002	0.27	0.83	25.4	0.74	15.6 (15.5±0.2)
0.004	0.53	0.83	26.0 (25.5)	0.74	16.0 (15.6±0.2)
0.02	2.67	0.83	25.7	0.74	15.7 (15.3±0.2)
0.04	5.34	0.83	25.3	0.71	14.9 (14.6±0.1)
0.4	53.67	0.82	24.3 (24.2)	0.66	13.1 (12.8±0.1)

[a]J_{cal} values in brackets were calculated from EQE measurements.
[b]PCE_{avg} values in brackets represent averages from 20 devices.

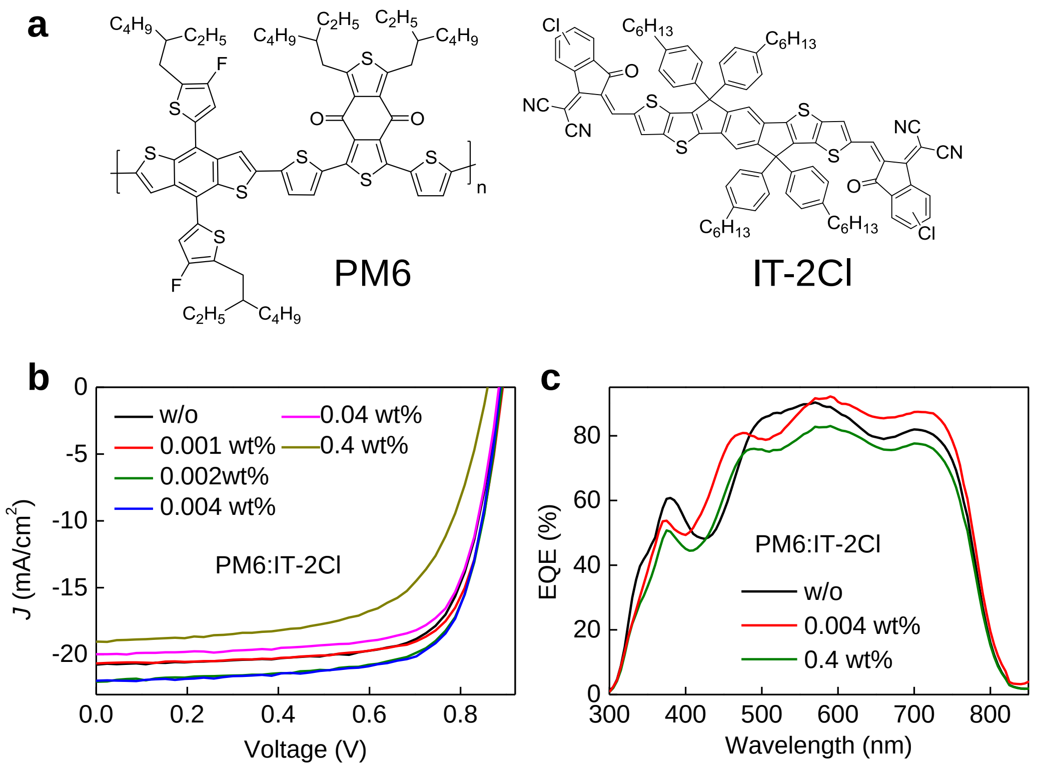

Figure S13. a) Chemical structures of PM6 and IT-2Cl. b) *J-V* curves and c) EQE curves of OPV cells based on PM6:IT-2Cl doped with different wt% of BV.

Table S7. Summary of photovoltaic operating parameters for PM6:IT-2Cl OPVs doped with different wt% of BV, measured under illumination of AM 1.5G (100 mW/cm^2).

BV [wt%]	BV [mol%]	V_{OC} [V]	J_{SC} (J_{cal})[a] [mA/cm^2]	FF	PCE_{max} (PCE_{avg})[b] [%]
0	0	0.89	20.8 (20.4)[a]	0.72	13.3 (13.0±0.1)[b]
0.001	0.13	0.89	20.1	0.74	13.6 (13.1±0.3)
0.002	0.26	0.89	22.0	0.72	14.2 (13.8±0.2)
0.004	0.52	0.89	22.0 (21.7)	0.73	14.3 (14.0±0.1)
0.04	5.15	0.88	19.6	0.74	12.8 (12.2±0.4)
0.4	51.52	0.86	19.1 (19.0)	0.64	10.4 (10.0±0.2)

[a]J_{cal} values in brackets were calculated from EQE measurements.
[b]PCE_{avg} values in brackets represent averages from 20 devices.

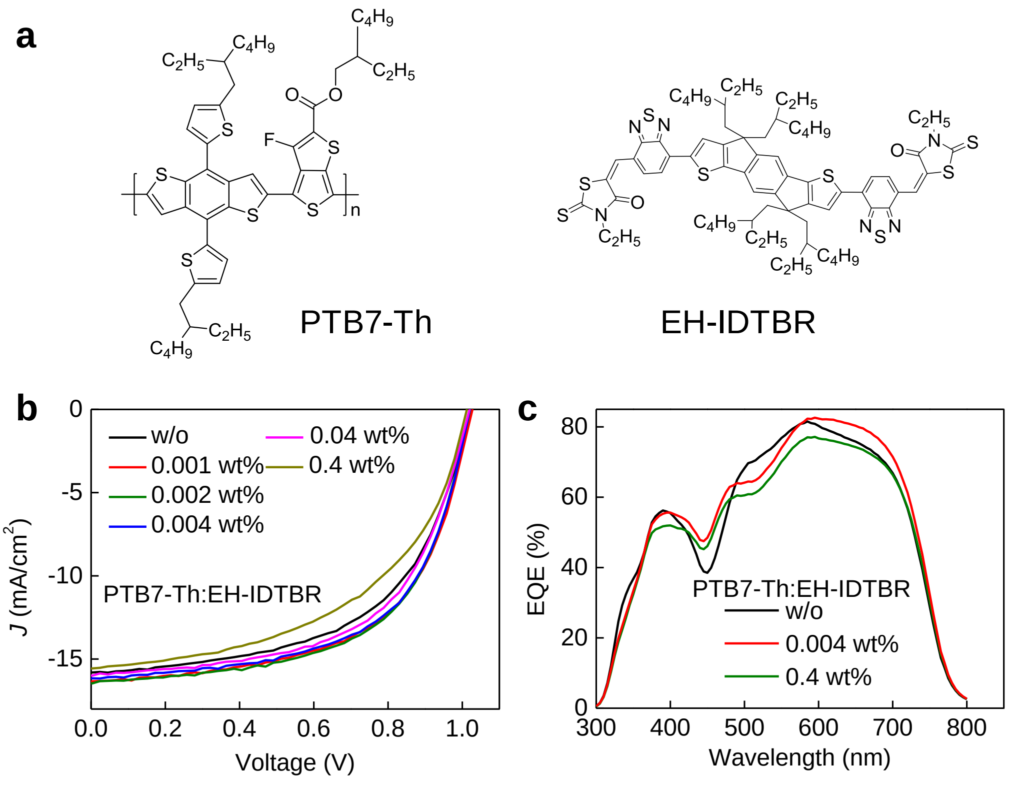

Figure S14. a) Chemical structures of PTB7-Th and EH-IDTBR. b) *J-V* curves and c) EQE curves of OPV cells based on PTB7-Th:EH-IDTBR doped with different weight ratios of BV.

Table S8. Summary of photovoltaic operating parameters for PTB7-Th:EH-IDTBR OPVs doped with different wt% of BV, measured under illumination of AM 1.5G (100 mW/cm^2).

BV [wt%]	BV [mol%]	V_{OC} [V]	J_{SC} (J_{cal})[a] [mA/cm^2]	FF	PCE$_{max}$ (PCE$_{avg}$)[b] [%]
0	0	1.02	15.8 (15.9)[a]	0.57	9.1 (8.7±0.2)[b]
0.001	0.10	1.02	16.3	0.61	9.8 (9.5±0.1)
0.002	0.20	1.02	16.4 (15.5)	0.60	9.9 (9.6±0.1)
0.004	0.39	1.02	16.1	0.60	9.8 (9.3±0.3)
0.04	3.88	1.02	16.0	0.58	9.5 (9.1±0.2)
0.4	38.78	1.01	15.6 (15.5)	0.52	8.1 (7.6±0.3)

[a]J_{cal} values in brackets were calculated from EQE measurements.
[b]PCE$_{avg}$ values in brackets represent averages from 20 devices.

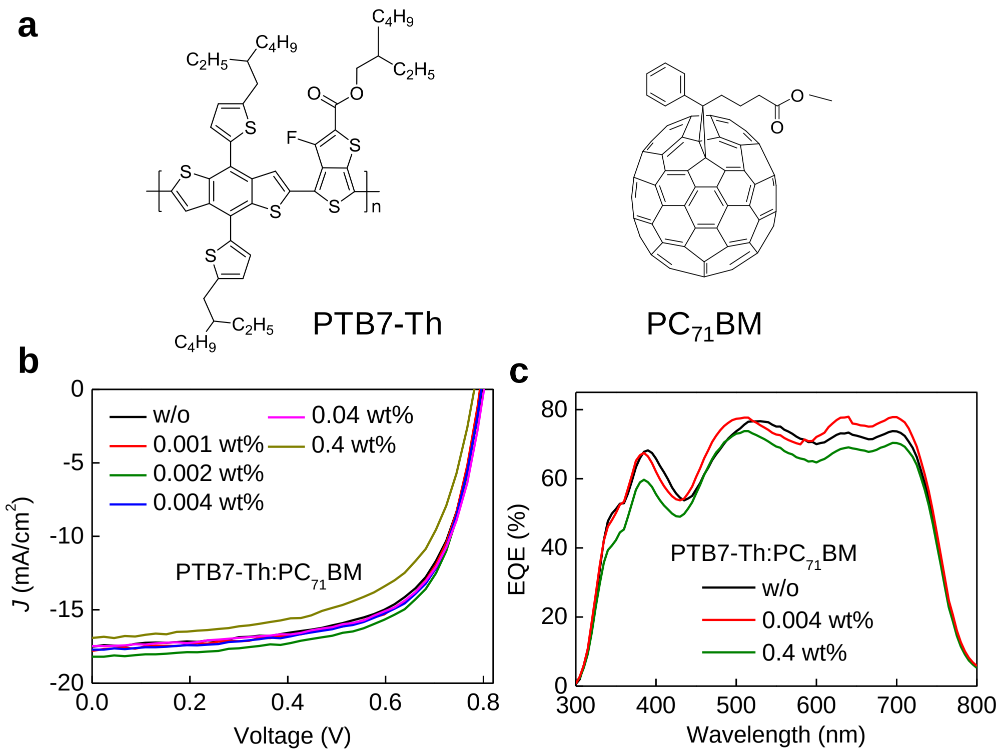

Figure S15. a) Chemical structures of PTB7-Th and PC$_{71}$BM. b) *J-V* curves and c) EQE curves of OPV cells based on PTB7-Th:PC$_{71}$BM doped with different wt% of BV.

Table S9. Summary of photovoltaic operating parameters for PTB7-Th:$PC_{71}BM$ OPVs doped with different wt% of BV, measured under illumination of AM 1.5G (100 mW/cm^2).

BV [wt%]	BV [mol%]	V_{OC} [V]	J_{SC} (J_{cal})[a] [mA/cm^2]	FF	PCE_{max} (PCE_{avg})[b] [%]
0	0	0.80	17.5 (17.0)[a]	0.65	9.0 (8.7±0.1)[b]
0.001	0.12	0.79	17.8	0.66	9.3 (9.2±0.1)
0.002	0.23	0.80	18.2 (17.9)	0.66	9.6 (9.4±0.1)
0.004	0.46	0.80	17.7	0.66	9.3 (8.9±0.2)
0.04	4.61	0.80	17.5	0.66	9.2 (8.9±0.1)
0.4	46.14	0.78	16.9 (16.4)	0.61	8.0 (7.9±0.1)

[a]J_{cal} values in brackets were calculated from EQE measurements.
[b]PCE_{avg} values in brackets represent averages from 20 devices.

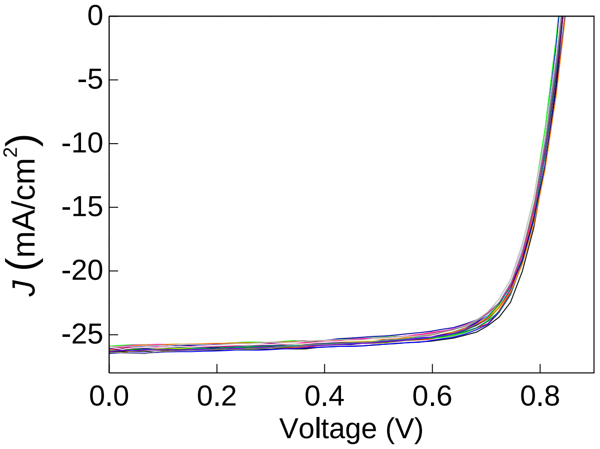

Figure S16. *J-V* curves of 20 devices based on PM6:Y6:$PC_{71}BM$ doped with 0.004 wt% of BV.

Table S10. Summary of photovoltaic operating parameters of 20 devices for PM6:Y6:$PC_{71}BM$ OPVs doped with 0.004 wt% of BV, measured under illumination of AM 1.5G (100 mW/cm^2)

Number	V_{OC} [V]	J_{SC} [mA/cm^2]	FF	PCE [%]
1	0.84	26.3	0.77	17.1
2	0.84	26.2	0.77	17.0
3	0.83	25.9	0.78	16.8
4	0.83	26.5	0.77	17.0
5	0.84	26.2	0.75	16.6
6	0.84	25.9	0.75	16.4
7	0.84	26.2	0.76	16.6
8	0.84	26.4	0.74	16.5
9	0.84	26.1	0.75	16.5
10	0.84	26.1	0.76	16.7
11	0.85	26.2	0.77	17.1
12	0.84	26.3	0.76	16.6
13	0.84	26.1	0.77	16.9
14	0.85	25.9	0.75	16.4
15	0.85	25.9	0.76	16.7
16	0.84	26.3	0.75	16.5
17	0.83	26.1	0.75	16.4
18	0.84	26.0	0.76	16.5
19	0.84	25.9	0.75	16.3
20	0.84	26.4	0.74	16.4

Reference

1. D.-H. Lee, M. Kang, D.-H. Lim, Y. Kim, J. Lee, D.-Y. Kim, K.-J. Baeg, *J. Mater. Chem. C* **2018**, 6, 5497.
